# Short telomere length in IPF lung associates with fibrotic lesions and predicts survival

**DOI:** 10.1371/journal.pone.0189467

**Published:** 2017-12-27

**Authors:** Reinier Snetselaar, Aernoud A. van Batenburg, Matthijs F. M. van Oosterhout, Karin M. Kazemier, Suzan M. Roothaan, Ton Peeters, Joanne J. van der Vis, Roel Goldschmeding, Jan C. Grutters, Coline H. M. van Moorsel

**Affiliations:** 1 Department of Pulmonology, St Antonius ILD Center of Excellence, St Antonius Hospital, Nieuwegein, the Netherlands; 2 Department of Pathology, St Antonius ILD Center of Excellence, St Antonius Hospital, Nieuwegein, the Netherlands; 3 Division of Heart and Lungs, University Medical Center Utrecht, Utrecht, the Netherlands; 4 Department of Pathology, University Medical Center Utrecht, Utrecht, the Netherlands; 5 Department of Clinical Chemistry, St Antonius ILD Center of Excellence, St Antonius Hospital, Nieuwegein, the Netherlands; Universitatsklinikum Freiburg, GERMANY

## Abstract

Telomere maintenance dysfunction has been implicated in the pathogenesis of Idiopathic Pulmonary Fibrosis (IPF). However, the mechanism of how telomere length is related to fibrosis in the lungs is unknown. Surgical lung biopsies of IPF patients typically show a heterogeneous pattern of non-fibrotic and fibrotic areas. Therefore, telomere length (TL) in both lung areas of patients with IPF and familial interstitial pneumonia was compared, specifically in alveolar type 2 (AT2) cells.

Fluorescent in situ hybridization was used to determine TL in non-fibrotic and fibrotic areas of 35 subjects. Monochrome multiplex quantitative polymerase chain reaction (MMqPCR) was used for 51 whole lung biopsies and blood TL measurements.

For sporadic IPF subjects, AT2 cell TL in non-fibrotic areas was 56% longer than in fibrotic areas. No such difference was observed in the surrounding lung cells. In subjects carrying a telomerase reverse transcriptase (*TERT)* mutation, AT2 cell TL was significantly shorter than in sporadic subjects. However, no difference in surrounding cell TL was observed between these subject groups. Finally, using biopsy MMqPCR TL measurements, it was determined that IPF subjects with shortest lung TL had a significantly worse survival than patients with long TL.

This study shows that shortening of telomeres critically affects AT2 cells in fibrotic areas, implying TL as a cause of fibrogenesis. Furthermore, short lung telomere length is associated with decreased survival.

## Introduction

Idiopathic pulmonary fibrosis (IPF) is a rare lung disease characterized by progressive fibrosis of lung parenchyma [1]. Patients with the disease have a median post-diagnostic survival of 2–5 years [2]. IPF can be both a sporadic and a familial disease. The familial form can be caused by mutations in surfactant related genes, or genes that influence telomere maintenance [3–10]. Analysis of familial IPF patients with mutations in telomerase reverse transcriptase (*TERT)* or telomerase RNA component (*TERC)* showed a diminished telomerase activity and prematurely shortened telomere length (TL) in blood leukocytes. Similar results were found in sporadic patients not carrying telomerase mutations, when compared to healthy controls [11–13]. It was also shown that TL of the lung alveolar type 2 (AT2) cells of IPF patients was shorter compared to controls [11]. Together, these findings indicate that telomere related pathology plays a role in both familial and sporadic IPF. However, it remains unknown whether the short TL in AT2 cells is related to fibrosis.

A contemporary view on the pathogenesis of IPF focuses on the role of AT2 cell during disease development [9,14–16]. Evidence for this can be found in patients diagnosed with a surfactant-related familial interstitial pneumonia (FIP). Since AT2 cells are the exclusive producers of surfactant protein-C, these cells are considered to be the precursor cells leading to pulmonary fibrosis [17]. Conversely, a link between mutations in telomerase related genes and the AT2 cell is not clear. In healthy lung tissue, the AT2 cell provides the regenerative capacity of the lung alveoli [18]. Faulty telomere maintenance could underlie an impaired proliferative capacity of the AT2 cells [19]. Recently it has been demonstrated that mice with telomere repeat binding factor 1 (TRF1)-deleted AT2 cells develop lung fibrosis and showed short telomeres in AT2 cells [20,21]. This might explain the human AT2 cell TL shortening in IPF, which could result in a similar response characterized by progressive fibrosis [22]. If telomere shortening plays a role in IPF disease development, it would be expected to occur primarily in AT2 cells.

IPF lungs show a patchy distribution of affected fibrotic and relatively preserved, non-fibrotic tissue [23,24]. This heterogeneous distribution allows for a comparison of TL between non-fibrotic and fibrotic tissue in a single surgical biopsy. In this study we investigated how the distribution of telomere shortening in lung tissue biopsies of patients is related to fibrotic remodeling of the tissue. We show that in sporadic IPF, AT2 TL was significantly longer in non-fibrotic areas than in fibrotic regions, thereby implicating telomere shortening as a cause of fibrotic remodeling of lung tissue in IPF. In addition, familial patients with a *TERT* mutation show significant shorter telomeres than in sporadic IPF. Furthermore, short whole biopsy telomere length in sporadic IPF patients is associated with worse survival.

## Material and methods

### Human subjects

In this study, 63 patients diagnosed with IPF at the St. Antonius ILD Center of Excellence Nieuwegein were included retrospectively (Table 1). In these patients, TL was measured in AT2 cells, whole lung biopsies and white blood cells. Patients were classified as either sporadic IPF (n = 39) or familial interstitial pneumonia (FIP) (n = 24). Diagnoses were based on the ATS/ERS/JRS/ALAT guidelines after multidisciplinary discussion [1,25]. The disease was designated as familial if two or more first-degree family members suffered from idiopathic interstitial pneumonia. FIP patients were screened on mutations in *TERT*, *TERC*, surfactant protein C (*SFTPC*), surfactant protein A2 (*SFTPA2*) exon 6 and TRF1-Interacting Nuclear Factor 2 (*TINF2*) exon 6. Based on these results, the FIP group was subdivided in two subgroups: patients that carried a mutation in *TERT*: FIP-TERT (n = 10, S1 Table) and patients that did not carry a known mutation in telomere related genes: FIP-nonTERT (n = 14). The latter subgroup included 3 patients with a *SFTPC* or *SFTPA2* mutation. Of the remaining 11 patients, no known pathogenic mutations were found. To assess lung function parameters, diffusing capacity of the lungs for carbon monoxide (DLCO) and forced vital capacity (FVC) data were collected within a 3-month window before or after diagnosis (n = 39). To cross reference results, a control group was formed using normal lung tissue obtained during post-mortem examination of five subjects not suffering from lung related pathology. Patient characteristics were retrieved from medical reports. The study was approved by the Medical research Ethics Committees United (MEC-U) of the St Antonius Hospital (approval number W14.056 and R05.08A). All patient data were anonymized.

**Table 1:** Patient group characteristics.

	IPF	FIP-TERT	FIP-nonTERT
Total n (% male)	39 (90%)	10 (80%)	14 (57%)
**Mean (SD)**			
Age at diagnosis in years	61 (10)	64 (7)	54 (12)
DLCO % predicted	47 (18)	47 (10)	43 (15)
FVC % predicted	69 (22)	85 (8)	63 (22)

IPF: idiopathic pulmonary fibrosis, FIP: familial interstitial pneumonia, SD: standard deviation, FVC: forced vital capacity, DLCO: diffusing capacity of the lungs for carbon monoxide

### Lung tissue

**Fig 1:**
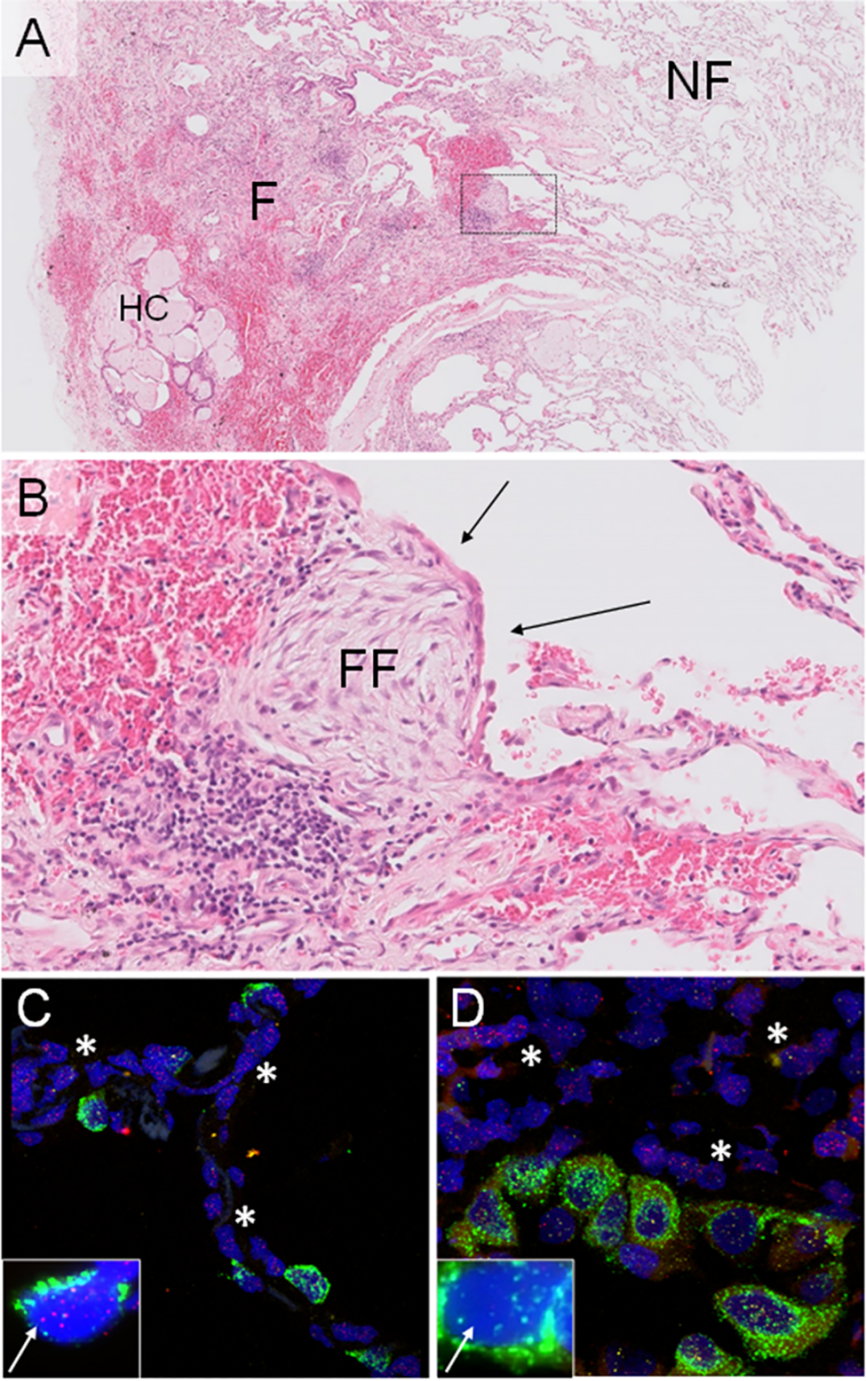
Images showing representative regions for non-fibrotic and fibrotic areas within one lung biopsy. (**A**) Low magnification image of H&E stained IPF lung tissue showing non-fibrotic (NF) and fibrotic (F) areas, and characteristic honeycombing (HC). (**B**) Enlarged boxed area of image **A** representing a fibrotic area in IPF lung biopsy. Characteristic fibroblast focus (FF) and alveolar type 2 (AT2) cell hyperplasia (black arrows) are highlighted. (**C,D**) Combined fluorescent images of AT2 and surrounding cells in non-fibrotic areas (**C**) and fibrotic areas (**D**) in IPF lung tissue. DNA in nucleus is displayed in blue (DAPI), pro-SPC in green and telomeres in red. Note the lower signal of AT2 cell (white arrows) telomere signal (red dots) in fibrotic areas (**D**) vs non-fibrotic (**C**) tissue, reflecting shorter telomeres in AT2 cells in fibrotic areas. Examples of pro-SPC negative surrounding cells are marked with an asterisk (*).

Residual lung tissue was obtained from biopsies carried out for diagnostic purposes and was fixed in formalin and embedded in paraffin (FFPE). Serial sections of 4 μm were cut. Non-fibrotic and fibrotic areas were identified on hematoxylin & eosin (H&E) stained sections (Fig 1A and 1B). All identifications were done by pathologists (MvO and SR), who are highly experienced in the field of interstitial lung diseases.

### Fluorescent *in situ* hybridization

After identification of fibrotic and non-fibrotic areas, the sequential section of the biopsy was used for a fluorescence *in situ* hybridization (FISH). Tissue slides were deparaffinized using a xylene series. Next, they were placed in H_2_O_2_ block buffer (1.5%), washed in Phosphate-buffered saline (PBS) and treated with Borax (1 mg/mL). For antigen retrieval, specimens were boiled in a citrate solution for 20 min (2.94 g/L, pH 6). Telomeres were labeled with a telomere-Cy3 PNA probe (Panagene, Daejeon, South-Korea) and pro-SPC (AB3786, 1/500, Merck Millipore, Darmstadt, Germany) was fluorescently labeled to identify AT2 cells (secondary antibody; A-11008, 1/300, Thermo Fisher Scientific, Waltham, MA, USA) (Fig 1C and 1D). Pro-SPC negative surrounding cells were used as reference. Surrounding cells were located within 2 cells of AT2 cells. Finally, DNA of samples was stained with 4',6-diamidino-2-phenylindole (DAPI, 25 μg/mL) and finished with Vectashield antifade mounting medium (Vector laboratories, Burlingame, CA, USA). Slides were stored at 4°C until analysis.

### Imaging and signal quantification

FISH-TL was measured using a method adopted from Meeker *et al*. [11,26,27]. Images were captured using a Fluorescence microscope (Leica DM 5500 B) at high magnification (100x). Per biopsy, up to 15 images were made per area. Z-stacking of 9 focal planes with 0.5μm intervals was used to maximize the coverage of cell nuclei. Total telomere (cy3) fluorescent signal was quantified per nucleus using the Telometer image analysis plugin (available at http://demarzolab.pathology.jhmi.edu/telometer/index.html) of ImageJ (http://rsb.info.nih.gov/ij/). To account for sub-optimal capturing of the nuclei caused by the cutting planes, total telomere signal was divided by the total DNA (DAPI) signal. All images were taken at fixed time points between 1 to 3 days after staining to circumvent data variability by DAPI fluorescence fading.

### MMqPCR for telomere length in FFPE tissue

DNA was isolated from FFPE tissue sections using an AllPrep DNA/RNA FFPE Kit (Qiagen, Hilden, Germany) according to manufacturer instructions. Slides were cut from sequential sections used for FISH. The paraffin was removed using paraffin dissolver (Macherey-Nagel, Düren, Germany). DNA was quantified using a Nanodrop (Thermo Fisher Scientific, Waltham, MA, USA) using an absorbance ratio of 260 and 280nm. Samples within a ratio of 1.8–2.0 were included. To measure whole lung biopsy and white blood TL, monochrome multiplex qPCR (MMqPCR) was performed as described earlier [13,28]. Because amplification of telomere and β-globin in FFPE DNA is delayed compared to blood derived DNA we adjusted cycle counts for all FFPE samples with -5 and -7 respectively. The relative TL for each sample was estimated from the ratio telomere repeat copy number (T) to a single human β-globin gene copy number (S) (T/S ratio), using standard curves from a serial dilution of a genomic DNA-pool [28]. Quadruplicate reactions were performed on a MyiQ™ Single-Color Real-Time PCRDetection System (Bio-Rad, Hercules, CA, USA) using iQ SYBR Green Supermix (Bio-Rad, Hercules, CA, USA). MMqPCR is proven to be a sensitive method to discriminate between patients with high and low TL [13].

### Statistics

Ratios were calculated for *non-fibrotic / fibrotic* and *AT2 cell / surrounding cell* comparisons. Values below 1 indicate shorter FISH-TL in non-fibrotic areas and AT2 cell respectively.

All analyses were performed using non-parametric statistical tests. Mann-Whitney and Wilcoxon signed ranked tests were used to compare TL. P-values for two-sided t-tests are shown. Correlations were determined using Spearman’s rank coefficient test. Survival analysis was done using Kaplan-Meier estimation. For statistical analysis IBM SPSS Statistics 22. (IBM Corp., Armonk, NY, USA) and GraphPad Prism 5 and 6 (GraphPad Software, San Diego, CA, USA) were used.

## Results

### DAPI is a valid measure to correct for total DNA per cell

For the FISH-TL measurements, DAPI was used to account for the total amount of DNA per cell. To verify whether DAPI staining was valid measure, we compared DAPI with a centromere FISH [29]. Similar results were found between both assays (n = 4, data not shown). Therefore we conclude that using DAPI as a counterstain is a valid method, as was found by Meeker and coworkers and Kropski and coworkers [27,30].

### Telomeres in non-fibrotic areas of sporadic IPF subjects are longer than in fibrotic areas

**Fig 2:**
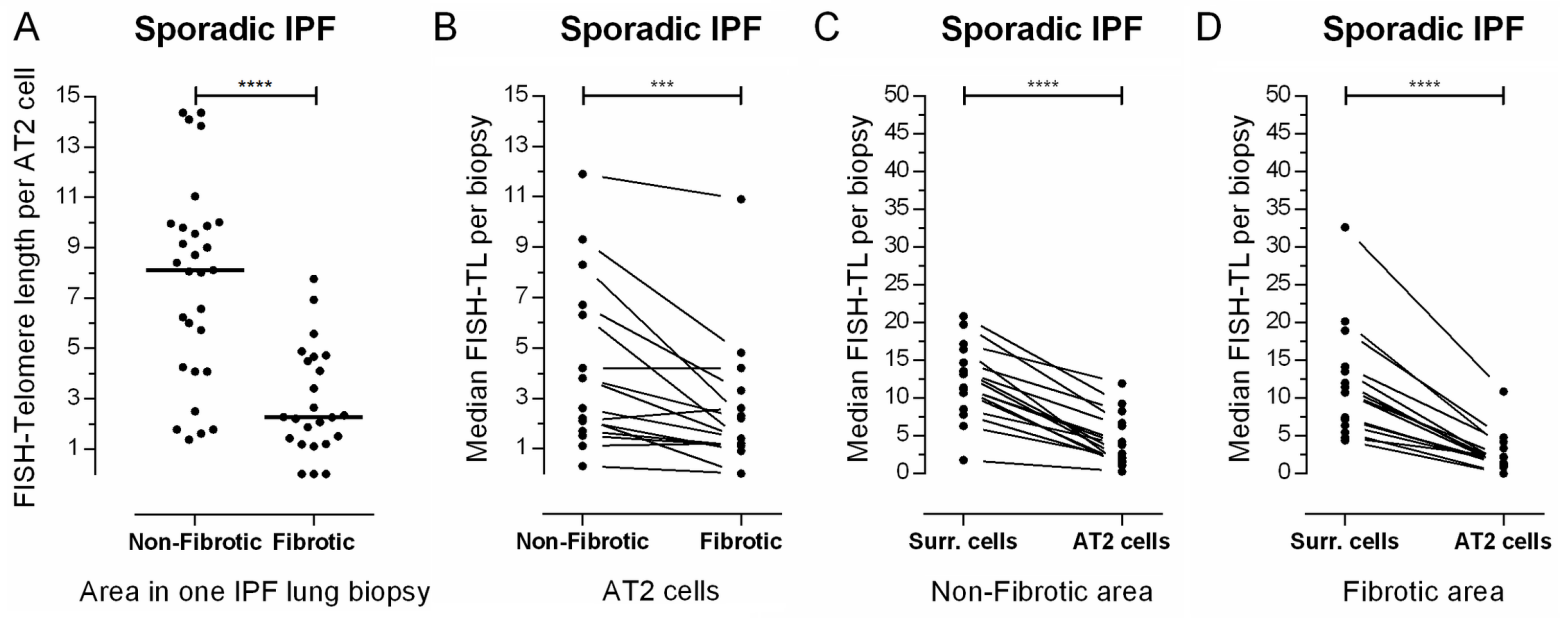
Telomere length in sporadic IPF lungs. (**A**) Representative example of telomere length per alveolar type 2 (AT2) cell for one sporadic IPF subject. Each dot represents one cell. Non-fibrotic AT2 FISH-TL is significantly higher compared to FISH-TL in fibrotic areas (p<0.0001). Bars represent medians and p-value is calculated using a Mann-Whitney test. (**B, C, D**) Median surrounding and AT2 cell FISH-TL in non-fibrotic and fibrotic areas for 16 sporadic IPF subjects. Each line connects, per subject, the FISH-TL of (**B**) AT2 cells in the non-fibrotic and fibrotic area or median FISH-TL differences between surrounding and AT2 cells in (**C**) non-fibrotic areas or (**D**) fibrotic areas. AT2 FISH-TL differences between non-fibrotic and fibrotic areas and between surrounding and AT2 cells were significant (2-tailed p<0.0006 and p<0.0001 respectively), which were indicated by asterisks (*** = p<0.001, **** = p<0.0001).

In order to investigate whether AT2 telomere shortening is related to fibrosis, we performed a FISH staining on FFPE material in a group of 16 sporadic IPF subjects. Median AT2 cell TL was significantly longer (p<0.001) in non-fibrotic areas compared to fibrotic areas (Fig 2), resulting in 2.24 times difference (FISH-TL ratio in Table 2). To get an idea of the general TL in non-fibrotic and fibrotic areas, we measured FISH-TL in pro-SPC negative surrounding cells. Here, no significant difference (p = 0.30, data not shown) was found between non-fibrotic and fibrotic areas (FISH-TL ratio: 1.15, Table 2).

**Table 2:** Median telomere length for non-fibrotic versus fibrotic areas per cohort.

		AT2 FISH-TL			Surrounding cell FISH-TL		
Subgroup	n	Non-Fibrotic (nf)	Fibrotic (f)	Ratio (nf/f)	Non-Fibrotic (nf)	Fibrotic (f)	Ratio (nf/f)
Controls	5	23.47	N/A	N/A	26.01	N/A	N/A
Spor IPF	16	3.22	1.44*	2.24	12.30	10.73	1.15
FIP-nonTERT	10	3.74	2.15*	1.74	12.60	7.46	1.68
FIP-TERT	9	1.00^#^	1.00	1.00	9.21	10.20	0.90

FISH: fluorescence in situ hybridization, TL: telomere length, AT2: alveolar type 2 cell, IPF: idiopathic pulmonary fibrosis, FIP: familial interstitial pneumonia.

Numbers indicate median telomere signal, of which ratios were calculated. Ratio (nf/f) = Non-fibrotic / Fibrotic, i.e. if ratio = 2 telomeres in non-fibrotic areas are two times longer than in fibrotic areas.

* = In non-fibrotic areas, AT2 FISH-TL is significantly longer than in fibrotic regions (spor IPF: p = 0.0006, FIP-nonTERT: p = 0.02).

# = AT2 FISH-TL in FIP-TERT is significantly shorter than in sporadic IPF (p = 0.02).

Fig 2 shows that FISH-TL is variable between subjects. To assess how FISH-TL diversity within subjects is distributed, the correlation between non-fibrotic and fibrotic FISH-TL was analyzed (data not shown). This resulted in a significant correlation for both AT2 (r = 0.855, p = 2∙10^−5^) and surrounding cells (r = 0.689, p = 0.003), indicating that FISH-TL variability among subjects is high, but correlates positively between non-fibrotic and fibrotic areas within a subject.

### Non-fibrotic and fibrotic AT2 cell telomere length in sporadic IPF subjects is shorter than in surrounding cells

Next, to elucidate further on telomere shortening in AT2 cells specifically, we compared telomere length of AT2 cells with surrounding cells. In non-fibrotic areas, the telomeres in AT2 cells were 4 times shorter than in surrounding cells (p<0.0001, Fig 2C and FISH-TL ratio: 0.26, Table 3). The difference was even larger in fibrotic areas: telomeres in AT2 cells were 8 times shorter than in surrounding cells (p<0.0001, Fig 2D and FISH-TL ratio: 0.13, Table 3). To place this in perspective, we determined the FISH-TL ratio between AT2 cells and surrounding cells in control subjects (n = 5). In controls no significant difference was found between AT2 and surrounding cells, indicating that under non-pathological conditions, AT2 cells do not have shortened telomeres (FISH-TL ratio: 0.90, Table 3).

**Table 3:** Median telomere length for alveolar type 2 (AT2) cells versus surrounding cells per cohort.

		Non-fibrotic area FISH-TL			Fibrotic area FISH-TL		
Subgroup	n	AT2	Surr.	Ratio (AT2/Surr.)	AT2	Surr.	Ratio (AT2/Surr.)
Controls	5	23.47	26.01	0.90	N/A	N/A	N/A
IPF	16	3.22	12.30*	0.26	1.44	10.73*	0.13
FIP-nonTERT	10	3.74	12.60*	0.29	2.15	7.46*	0.29
FIP-TERT	9	1.00^#^	9.21*	0.11	1.00	10.20*	0.10

FISH: fluorescence in situ hybridization, TL: telomere length, AT2: alveolar type 2 cell, IPF: idiopathic pulmonary fibrosis, FIP: familial interstitial pneumonia, surr.: pro-SPC negative surrounding cells. Numbers indicate median telomere signal, of which ratios were calculated. Ratio (AT2/Surr.) = *AT2 cell / surrounding cell*, i.e. if ratio = 2 telomeres in AT2 cells are two times longer than in surrounding cells.

# = AT2 FISH-TL in FIP-TERT is significantly shorter than in sporadic IPF (p = 0.02).

* = In both non-fibrotic and fibrotic areas, AT2 FISH-TL is significantly shorter than in surrounding cells (spor IPF: p < 0.0001, FIP-nonTERT and FIP-TERT: p < 0.01).

### Lung telomere length in Familial Interstitial Pneumonias: TERT

**Fig 3:**
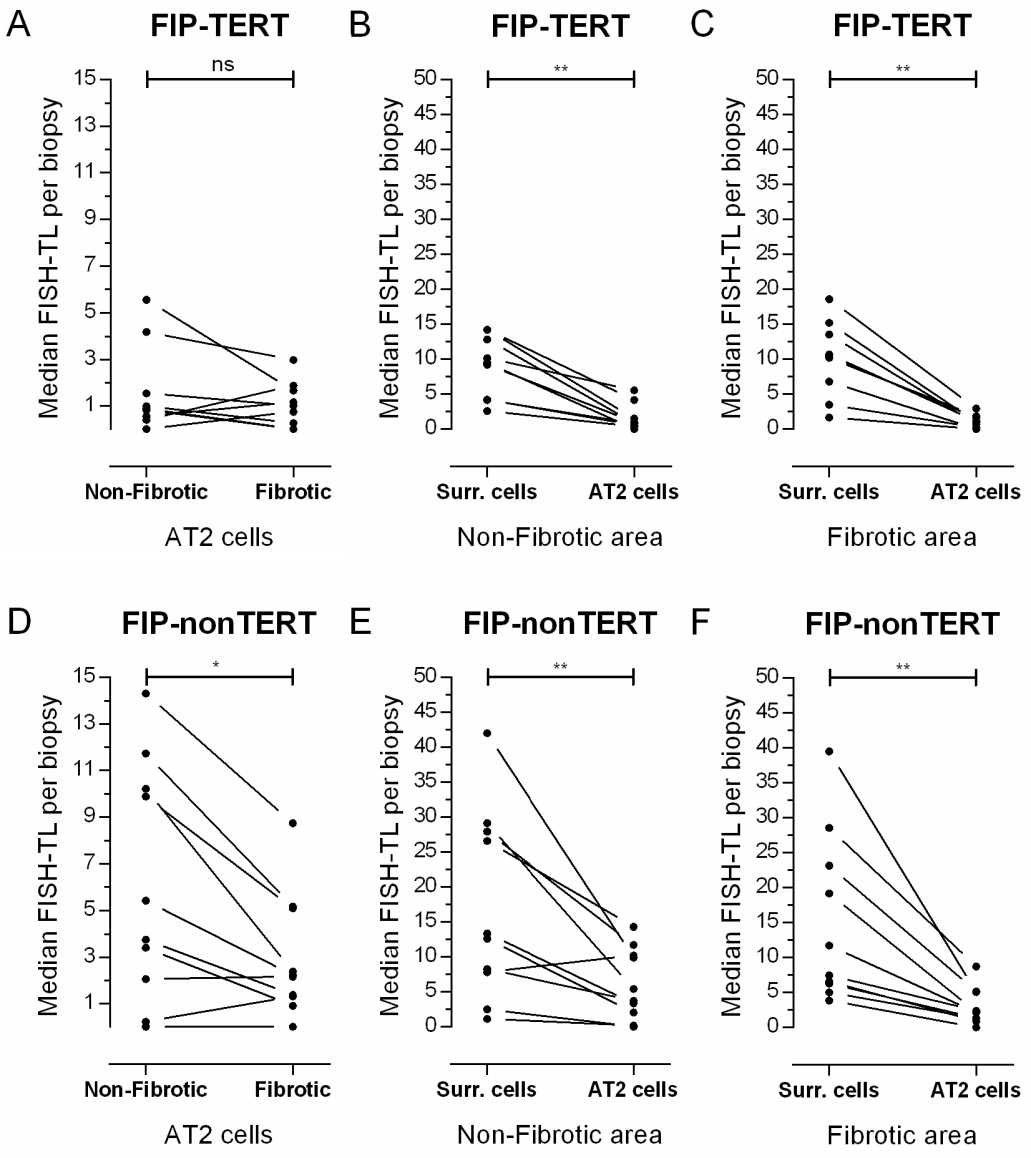
FISH telomere length in lungs of FIP-TERT and FIP-nonTERT subjects. (**A**) Median FISH-TL in alveolar type 2 (AT2) cells in non-fibrotic and fibrotic areas of 9 FIP-TERT subjects. Telomeres are generally short and no significant difference between areas was observed (2-tailed, p = 0.36). (**B, C**) Median FISH-TL of same subjects as in figure **A**, showing differences between surrounding and AT2 cells in (**B**) non-fibrotic and (**C**) fibrotic areas. (**D)** Median FISH-TL in alveolar type 2 (AT2) cells in non-fibrotic and fibrotic areas of 10 FIP-nonTERT subjects. Non-fibrotic AT2 FISH-TL is significantly higher compared to FISH-TL in fibrotic areas (2-tailed, p = 0.02). (**E, F**) Median FISH-TL of same subjects as in figure **D**, showing differences between surrounding and AT2 cells in (**E**) non-fibrotic and (**F**) fibrotic areas. Asterisks indicate significant differences calculated by Wilcoxon matched-pairs signed rank analysis (* = p<0.05, ** = p<0.01).

To investigate TL differences between sporadic IPF subjects and subjects with an established telomere syndrome, FISH-TL was determined in *TERT* mutation carriers (FIP-TERT). FIP-TERT showed no difference in AT2 FISH-TL between non-fibrotic and fibrotic areas (p = 0.36, Fig 3A). However, AT2 FISH-TL was substantially shorter in non-fibrotic areas compared to sporadic IPF (median 1.00 vs 3.22, p = 0.02, Table 2). In surrounding cells FISH-TL was concordant between FIP-TERT and sporadic IPF in both areas. FISH-TL in AT2 cells was significantly shorter than in surrounding cells in both non-fibrotic and fibrotic areas (p<0.01, Fig 3B and 3C). These data show that AT2 TL distribution between non-fibrotic and fibrotic tissue in FIP-TERT differs from sporadic IPF subjects, underlining the effect of a defective telomerase enzyme.

### Lung telomere length in Familial Interstitial Pneumonias: NonTERT

Next, we analyzed the familial subjects, who did not carry a known telomere related mutation (FIP-nonTERT). For these subjects, FISH-TL patterns were the same as in the sporadic IPF group; non-fibrotic AT2 cell FISH-TL was 1.74 times longer than fibrotic AT2 cells (p = 0.02, Fig 3D, Table 2). Furthermore, in both non-fibrotic and fibrotic areas the AT2 cell FISH-TL was 3.5 times shorter than surrounding cells (p<0.01, Fig 3E and 3F, FISH-TL ratio: 0.29, Table 3). Compared to FIP-TERT, FIP-nonTERT subjects had similar AT2 FISH-TL in non-fibrotic areas (p = 0.081).

In order to get an overview, we compared the sporadic IPF, FIP-TERT and FIP-nonTERT subject groups. All data is summarized in Tables 2 and 3. No statistical age differences were found between groups. Looking specifically at fibrotic areas, AT2 FISH-TL was not significantly different (p = 0.16) between subject groups. In non-fibrotic areas a significant difference in AT2 cell FISH-TL was observed between FIP-TERT and sporadic IPF subjects (p = 0.02) and a trend between FIP-TERT and FIP-nonTERT subjects (p = 0.081). These data indicate that shortest AT2 telomeres in non-fibrotic areas were found in FIP-TERT subjects. In addition, no significant difference in surrounding cells FISH-TL was found between the three subgroups in either area (p = 0.67), suggesting that in all subject groups TL shortening is most evident in AT2 cells.

### Telomere length by MMqPCR: Lung

**Fig 4:**
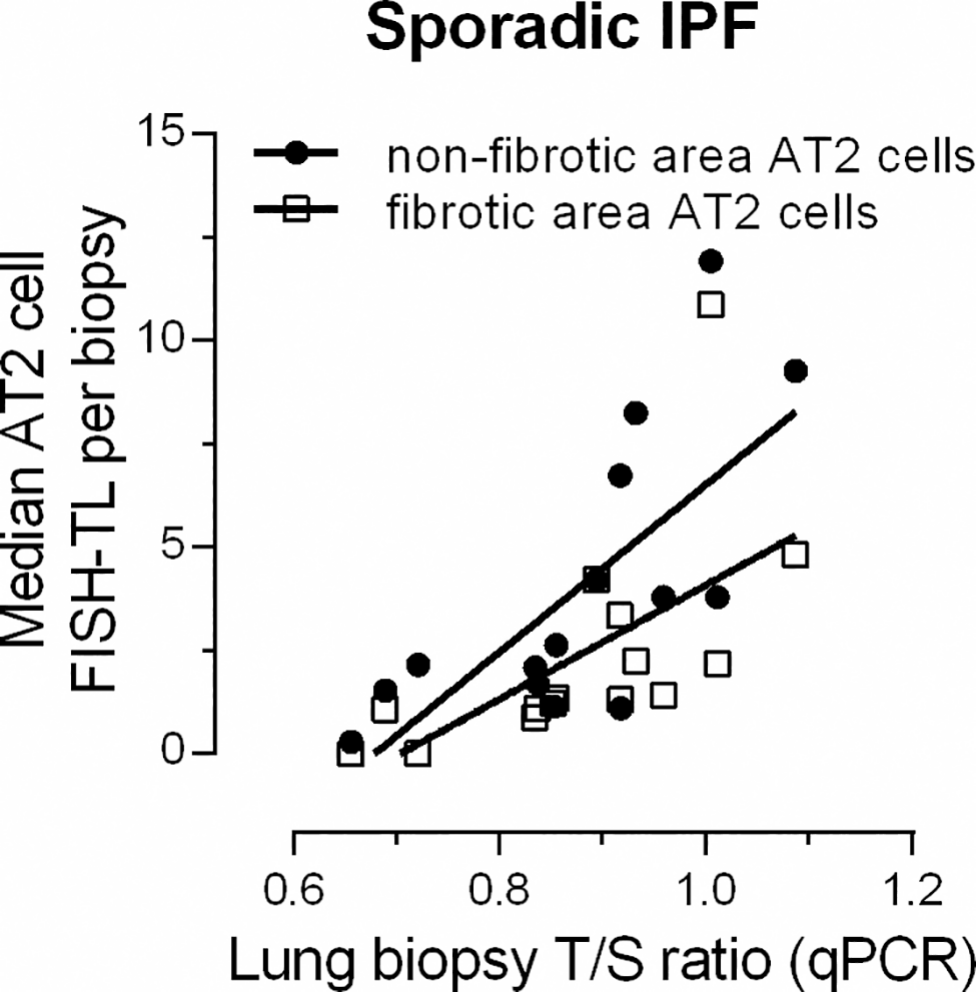
Correlation between FISH-TL and biopsy T/S in IPF subjects. Correlation (Spearman) between FISH-TL measured in alveolar type 2 (AT2) cells and biopsy T/S measured by MMqPCR (n = 15) in non-fibrotic areas (black dots) and fibrotic areas (open squares). Significant correlations were established between biopsy T/S and AT2 cell FISH-TL in non-fibrotic (r^2^ = 0.53, p = 0.002) and fibrotic (r^2^ = 0.73, p<0.0001) areas.

To test whether whole lung biopsy TL as measured by MMqPCR (biopsy T/S) correlates with FISH-TL of AT2 cells, we extracted DNA from biopsy sections and performed MMqPCR as described by Cawthon *et al*. (IPF n = 15, FIP-TERT n = 9, FIP-nonTERT n = 10) [28]. In sporadic IPF subjects, a significant positive correlation was found between biopsy T/S and AT2 FISH-TL in both non-fibrotic (r^2^ = 0.53, p = 0.002) and fibrotic (r^2^ = 0.73, p<0.0001) areas (Fig 4). No correlations were found in the FIP-TERT and FIP-nonTERT (data not shown).

### Telomere length by MMqPCR: Blood

**Fig 5:**
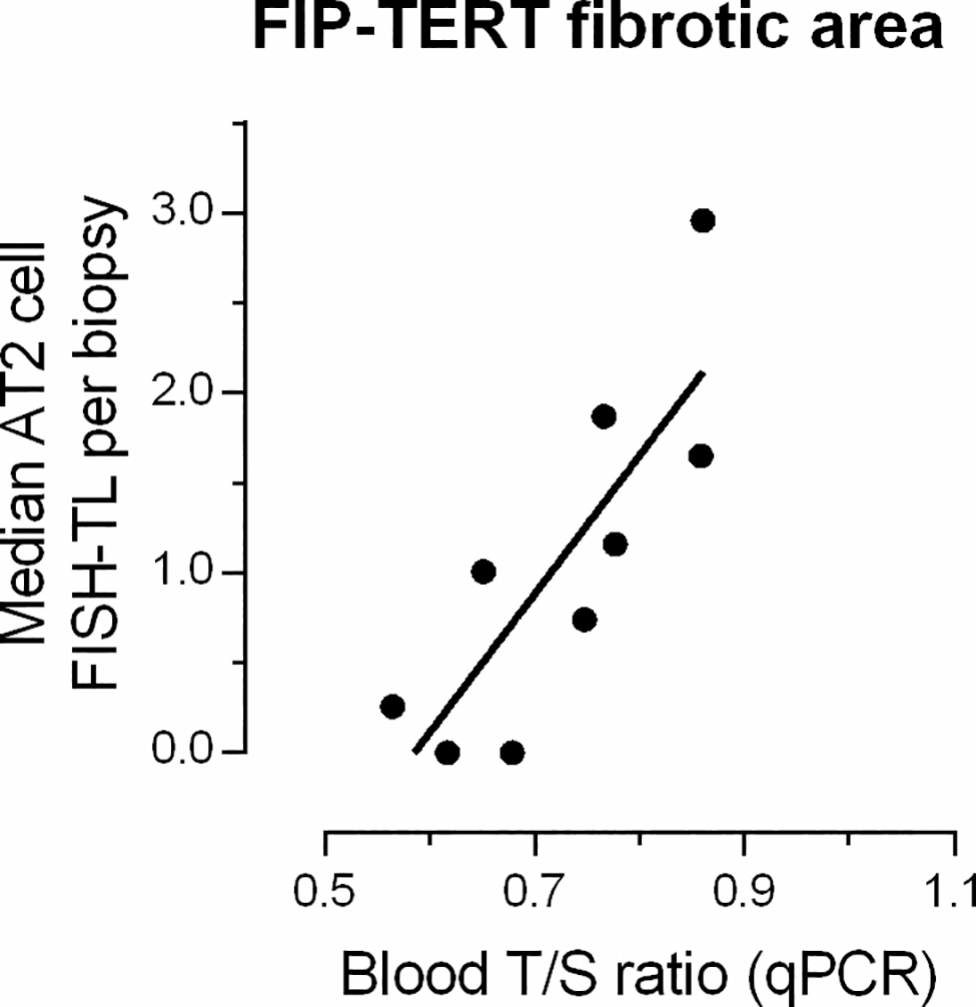
Correlation between FISH-TL and MMqPCR leukocyte TL (blood T/S) in FIP-TERT fibrotic areas. Positive FIP-TERT correlation (Spearman) between alveolar type 2 (AT2) cell FISH-TL in fibrotic areas and MMqPCR blood T/S (n = 9, r^2^ = 0.67, p = 0.007).

**Fig 6:**
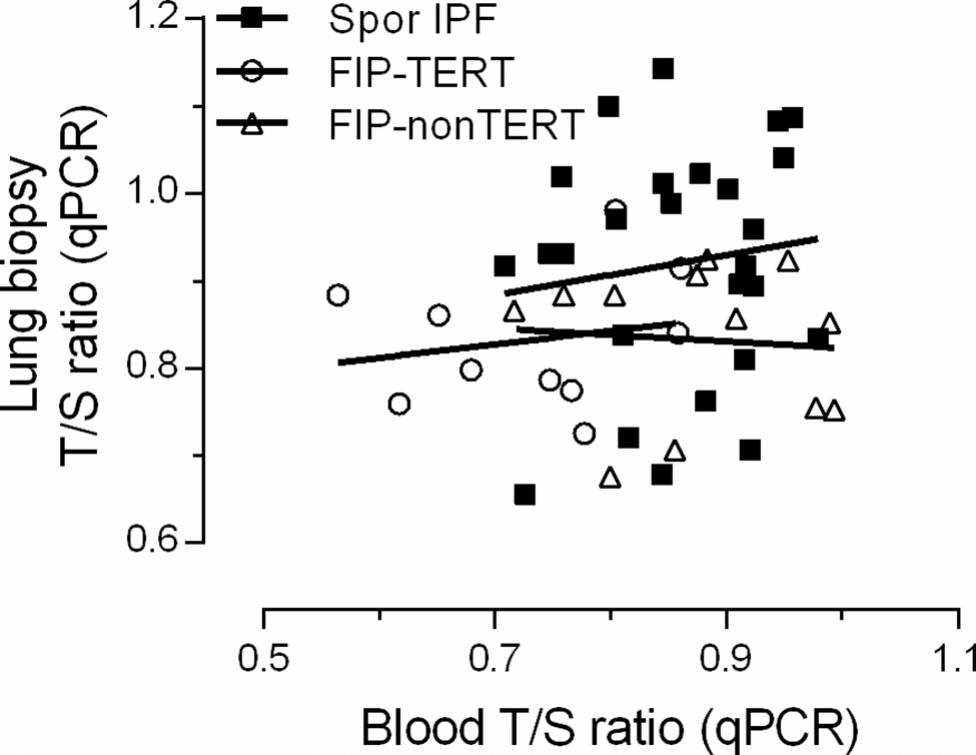
Correlations between MMqPCR measurements biopsy T/S and blood T/S in IPF. No correlation between biopsy T/S and blood T/S was observed in sporadic IPF (n = 26), FIP-nonTERT (n = 12) and FIP-TERT (n = 10) subjects.

We tested for a correlation between peripheral white blood cell TL measured with MMqPCR (blood T/S) and FISH-TL of lung tissue. We found no significant correlation between AT2 FISH-TL in fibrotic areas and blood T/S, except in FIP-TERT subjects. (r^2^ = 0.67, p = 0.007) (Fig 5). Also no significant correlation was found between MMqPCR measurements biopsy T/S and blood T/S (sporadic IPF; n = 26, FIP-nonTERT; n = 12 and FIP-TERT; n = 10, Fig 6).

### Short telomeres are associated with worse survival

**Fig 7:**
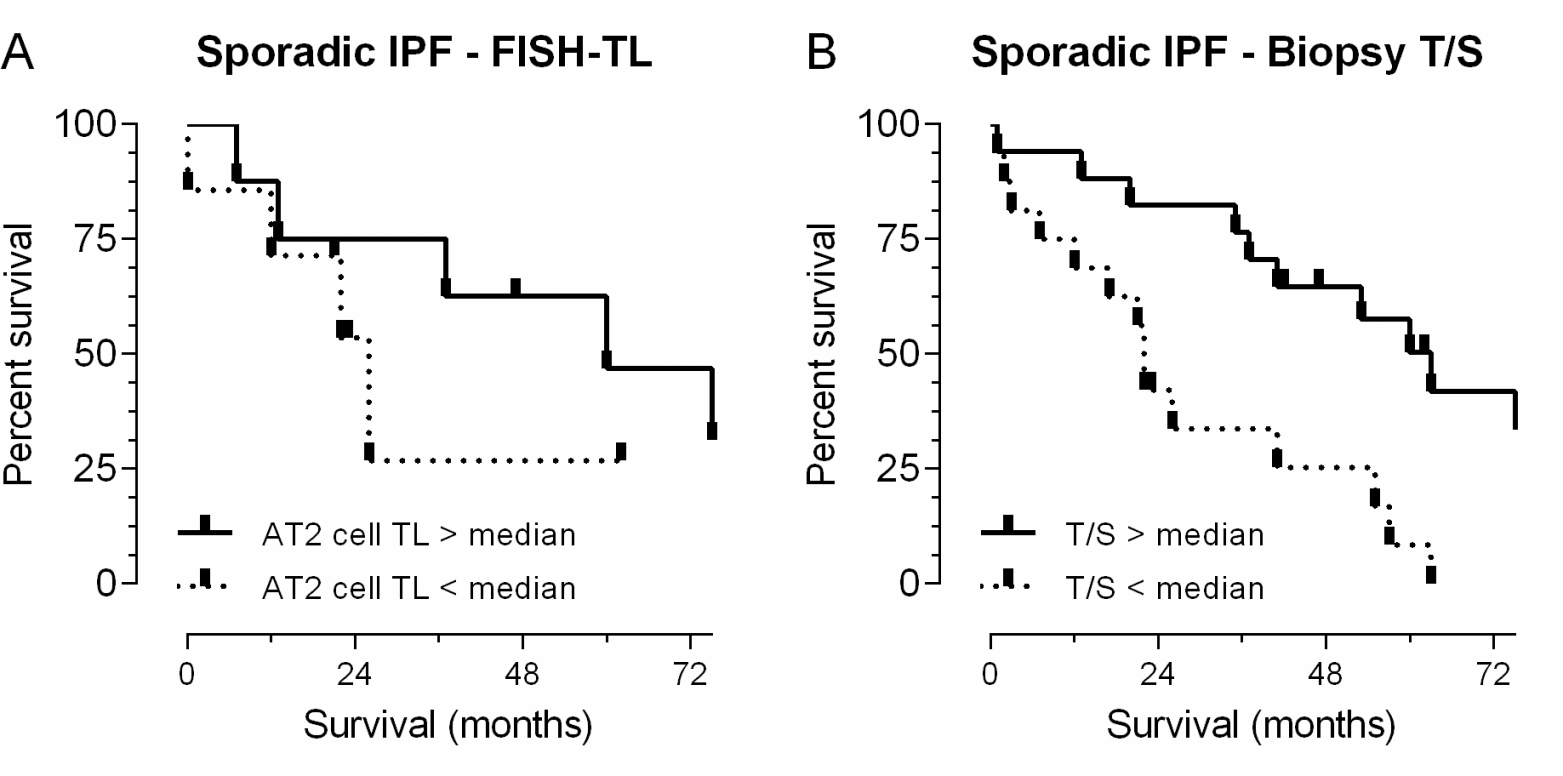
Survival of IPF patients. (**A**) FISH-TL on lung biopsy; Kaplan-Meier curve of 15 IPF patients showing a median survival of 60 months for patients with AT2 cell FISH-TL in non-fibrotic areas above median TL (solid line) and a median survival of 26 months for patients with AT2 cell TL below median TL (dotted line). The difference in median survival is 34 months (p = 0.353, Mantel-Cox). (**B**) MMqPCR on lung biopsy; Kaplan-Meier curve of 34 IPF patients showing a median survival of 63 months for IPF patients with a lung biopsy MMqPCR T/S ratio above the median (solid line) and a median survival of 22 months for patients with a lung biopsy MMqPCR T/S ratio below the median T/S (dotted line). The significant difference in median survival is 41 months (p = 0.003, Mantel-Cox). Deceased n = 27, lung transplantation n = 4, still alive n = 3.

In the literature, short peripheral leukocyte telomere length has been associated with worse survival time in IPF [31,32]. Here we investigated whether a shorter survival time is similarly associated with FISH-TL. We showed that in non-fibrotic regions AT2 FISH-TL variability between IPF subjects was substantial (Figs 2 and 3). To test whether this variability is associated with survival we divided this group (n = 15) at the median AT2 cell TL. Survival was calculated from date of biopsy until death (n = 9) or censoring of the patient (lung transplantation n = 3, still alive n = 3). Kaplan Meier survival analysis showed that patients with shortest AT2 cell TL had a lower median survival rate than patients with longest AT2 cell TL (26 months vs 60 months, p = 0.353, Fig 7A). Because lack of significance could be caused by underpowered analysis and because a significant positive correlation between biopsy T/S and AT2 FISH-TL was established above, we also performed a survival analysis using biopsy T/S (n = 34). Dividing the patient group at the median T/S, a significant difference in survival rate (p = 0.003) was found. Patients with a low T/S had decreased median survival of 22 months and lived 41 months shorter than patients with high T/S (Fig 7B). There were no significant differences in mean age at date of biopsy between the group with TL above median and the group with TL below median in either AT2 FISH-TL and biopsy T/S analyses.

## Discussion

In this study, we found that telomere shortening is predominantly observed in AT2 cells and associates with fibrotic lesions in IPF lung biopsies (Figs 2 and 3). Furthermore, patients with short lung telomeres had significantly worse survival than patients with longer telomeres (Fig 7).

Telomere shortening in AT2 cells is in accordance with two experimental observations where mice with telomere repeat binding factor 1 (TRF1)-deleted AT2 cells develop lung fibrosis and present short telomeres in AT2 cells [20,21].

To date, no study investigated the association between AT2 TL and the characteristic non-fibrotic and fibrotic regions in human IPF lungs with (FIP-TERT) or without a mutation in the telomerase gene. In fibrotic areas of sporadic IPF and FIP-TERT subjects we found that AT2 cells contain short telomeres, confirming results of Alder and coworkers [11]. However, in contrast to sporadic IPF, FIP-TERT AT2 telomere length was equally short in non-fibrotic areas and fibrotic areas (p = 0.36, Fig 3A). This suggests that patients with a telomere mutation are born with “aged-short telomere containing” lungs or that their lungs age at an increased rate. The latter is most probable because we also found that telomere length of surrounding cells was similar in FIP-TERT lungs, sporadic IPF and FIP-nonTERT lungs.

The pivotal role of AT2 cells in IPF pathogenesis is highly supported by the discovery of disease causing mutations in *SFTPC*. AT2 cells exclusively produce Surfactant protein C [33–35]. Additionally, besides producing and regulating surfactant fluid in the alveoli, AT2 cells are the progenitor cells that can differentiate into the gas diffusing AT1 cells [36]. This regenerative function of AT2 cells requires active telomere maintenance [37]. Indeed, telomerase has been shown to be active and upregulated in subpopulations of rat AT2 cells after hypoxic injury [37–39]. Additionally, literature postulated that shortened telomere length in blood is a risk factor for sporadic IPF and FIP-TERT subjects [6,7,11–13,31].

To clinically target the potential pathogenic AT2 cells in fibrosis, it might be feasible in the near future to introduce AT2 cell transplantation. It has been shown in the literature that AT2 cell transplantation is safe and well tolerated in IPF patients [40]. However, the therapeutic effect on fibrogenesis still has to be elucidated.

In contrast to AT2 cells, no difference was observed in TL of surrounding cells between non-fibrotic and fibrotic areas. Additionally, in healthy lung tissue no difference was found between AT2 and surrounding cells (Table 2). This also suggests that in pulmonary fibrosis, anomalous telomere shortening primarily affects AT2 cells.

In fibrotic areas, no differences were found in AT2 TL (p = 0.16) between subject groups. This could suggest that a critically short TL threshold must be reached for the development of fibrosis.

In general, critically short telomeres eventually lead to cell senescence or apoptosis, limiting the regenerative capacity of tissue [41,42]. Furthermore, mice with AT2 cell dysfunctional telomeres showed impaired response to induced injury [9]. In IPF an increase of apoptosis and senescence signaling has been reported in fibrotic areas. This might be the causal link between telomere shortening and IPF onset [43–46]. Moreover, mice with critically AT2 short telomeres are linked to elevated levels of pro-fibrotic TGF- β1 release [20], which also may lead to the development of lung fibrosis [47].

Next, we showed that measuring TL using MMqPCR on DNA extracted from whole lung biopsy sections of sporadic IPF can replace TL measured by FISH (Fig 4). This MMqPCR technique allows a time and labor efficient method of estimating lung telomere length and allowed us to efficiently double the sample size for survival analysis. Using whole lung biopsies, a significant negative survival was found in sporadic IPF patients with short TL (Fig 7B). The association is in accordance with previous studies reporting short leukocyte telomere length (measured by MMqPCR) to negatively influence survival [31,32].

There are conflicting reports concerning the correlation between blood TL and lung FISH-TL [11,26]. In our study, TL measured in blood cells did not correlate with FISH-TL in the lung (data not shown). This is in concordance with a report by Kropski et al [26]. However, in FIP-TERT patients we did find a correlation between TL in blood and in fibrotic area AT2 cells (Fig 5). This suggest that only in the presence of a *TERT* mutation telomeres in peripheral leukocytes and AT2 cells in fibrotic areas have comparable rates of shortening. Germ line *TERT* mutations affect all cells in the body and therefore TL in lung and all other cells are linked. Because the association between blood and lung telomere length is absent in sporadic patients we argue that telomere shortening in sporadic IPF patients is partly determined by the patient’s genetic constitution (explaining the shortened blood telomeres) and partly by lung specific factors (explaining the absence of a correlation between blood and lung telomere length). This is in accordance with the second hit theory in IPF, which implies that besides telomere shortening a local second hit, like a virus or smoking, might be responsible for elevated cell stress and development of fibrosis [16,48].

Given the influence of biopsy T/S on survival it might be useful to incorporate lung TL as a prognostic molecular biomarker in the interpretation and stratification of ongoing and future clinical trials. However, since the risk of complications of surgical lung biopsies is high [49,50] and diagnosis in IPF is often based on typical radiographic pattern of usual interstitial pneumonia, future studies will need to focus on less invasive methods to assess TL.

Strength of this study comprises the inclusion of three types of IPF cohorts; sporadic IPF, FIP-TERT and FIP-nonTERT. Comparison of TL between these groups is novel in this field of research. Furthermore, the comparison of fibrotic and non-fibrotic areas in one biopsy make the result independent of inter-assay differences. However, there are also some limitations in this study. The FIP-nonTERT group was chosen from familial patients with no telomere-related gene mutations. Screening was performed for *TERT*, *TERC*, *SFTPC*, *SFTPA2* exon 6 and *TINF2* exon 6. Other previously described IPF-related mutations, e.g. in dyskeratosis congenital 1 (*DKC1*), regulator of telomere elongation helicase 1 (*RTEL1*) and Poly(A)-specific ribonuclease (*PARN*) were not tested [8,30]. Therefore, we cannot exclude the presence of telomere-related genes in the FIP-nonTERT cohort.

In conclusion, this study shows that shortest telomeres are found in AT2 cells in fibrotic areas of IPF lung. Furthermore we show that short telomeres associate with shorter survival time. This provides new evidence for a critical role of short AT2 cell TL in the pathogenesis of IPF and maintenance of telomere length as a target for therapy.

## Supporting information

S1 Table Specific mutations carried by the FIP-TERT subjects in this study. (DOCX)

S1 Dataset Containing the raw data used in this study. (XLSX)
