# Supplementary material for: Short telomere length in IPF lung associates with fibrotic lesions and predicts survival
Source: PLoS One. 2017 Dec 27;12(12):e0189467. doi: 10.1371/journal.pone.0189467 (PMC5744955; doi:10.1371/journal.pone.0189467)
Supplement: S1 Table — (DOCX) [file pone.0189467.s001.docx]

| **cDNA Position** | **Variant name** | **SIFT*** | **PolyPhen-2^#^** | **Allele frequency (Exac)** | **Allele frequency (1000G)** | **Reference no.** | |
| --- | --- | --- | --- | --- | --- | --- | --- |
| c.455T>A | L152Q | D | D | not found | not found | NA | |
| c.1584T>G | C528W | T | P | not found | not found | NA | |
| c.1698_1700delCAC | T567del | NA | NA | not found | not found | NA | |
| c.2005C>T | R669W | D | B | 0.00006376 | not found | rs372140951 | |
| c.2011C>T | R671W | D | P | 0.00006342^ | not found | NA | |
| c.2303A>T | D768V | D | D | not found | not found | NA | |
| c.2406C>G | S802R | D | D | not found | not found | NA | |
| c.2146G>A | A716T | D | D | not found | not found | rs387907249 | |
| c.2701C>T | R901W | T | P | not found | not found | rs199422304^+^ | |
| c.2701C>T | R901W | T | P | not found | not found | rs199422304^+^ | |
| *SIFT prediction: Damaging (D) <=0,05; Tolerated (T) >0,05 | | | |  |  |  | |
| ^#^PolyPhen-2 HumVar analysis (% = False Positive Rate): Damaging (D) <=10%; Possibly damaging (P) >10% and <=20%; Benign (B) >20%. | | | | | | |  |
| ^Variant is associated with pulmonary fibrosis [1] | | | | | | |  |
| ^+^Variant is described in a patient with Hoyeraal-Hreidarsson syndrome, which is characterized by short telomeres [2]. | | | | | | |  |

**S1 Table. Specific mutations carried by the FIP-TERT subjects in this study**

**References**

[1]Diaz de Leon A, Cronkhite JT, Katzenstein AL, Godwin JD, Raghu G, Glazer CS, et al. Telomere lengths, pulmonary fibrosis and telomerase (TERT) mutations PLoS One 2010 May 19;5(5):e10680.

[2]Marrone A, Walne A, Tamary H, Masunari Y, Kirwan M, Beswick R, et al. Telomerase reverse-transcriptase homozygous mutations in autosomal recessive dyskeratosis congenita and Hoyeraal-Hreidarsson syndrome Blood 2007 Dec 15;110(13):4198-4205.
